# Understanding parental stress among parents of children with Paediatric Acute-onset Neuropsychiatric Syndrome (PANS) in Sweden

**DOI:** 10.1080/17482631.2022.2080906

**Published:** 2022-05-26

**Authors:** Noam Ringer, Lise Roll-Pettersson

**Affiliations:** Department of Special Education, Stockholm University, Stockholm, Sweden

**Keywords:** Paediatric Acute-onset neuropsychiatric syndrome, PANS, PANDAS, OCD, parental stress, coping, mental disorder, qualitative research, transactional theory, constructivist grounded theory

## Abstract

**Purpose:**

Paediatric Acute-onset Neuropsychiatric Syndrome (PANS) is a relatively new diagnosis characterized by an abrupt and dramatic onset of obsessive-compulsive disorder (OCD), together with neuropsychiatric symptoms. Very little research has been done to understand the experience of being a parent of a child with PANS. The current study aimed to explore aspects related to parental stress in parents of children with PANS.

**Method:**

The study employed in-depth semi-structured individual interviews with 13 parents of children diagnosed with PANS. Parents were recruited via an announcement on the websites of patient organizations, and in waiting rooms at child medical clinics. An inductive qualitative content analysis approach was used as a guide for analysis of data.

**Results:**

The analysis of interviews identified five categories of parents’ experiences of stress related to: (1) being effected by the symptoms; (2) experiencing the symptoms over and over again; (3) having no control; (4) obtaining medical treatment is challenging; and (5) managing problems. The results are discussed in relation to the Transactional Theory of Stress and Coping.

**Conclusions:**

the study illuminates how parents’ perceptions of the child’s symptoms, parents’ strategies for managing problems, as well as experiences related to healthcare providers, may increase or decrease parental stress.

## Introduction

Paediatric Acute-onset Neuropsychiatric Syndrome (PANS) is a medical condition characterized by an abrupt and dramatic onset of obsessive-compulsive disorder (OCD) symptoms and/or severely restricted food intake, together with neuropsychiatric symptoms such as anxiety, mood dysregulation, irritability, aggression, oppositionality, behavioural regression, cognitive deterioration, sensory or motor abnormalities, and somatic symptoms (Chang et al., 2015). The abrupt and rapid onset of symptoms that characterizes PANS is often described in terms of a “flare” (Gromark et al., 2021; Leon et al., 2018). The prevalence of the condition is still not clear, and researchers illuminate the need for large-scale epidemiologic studies to characterize the incidence, prevalence, and demographics of the syndrome (Swedo et al., 2012). However, a study of a sample of paediatric patients diagnosed with OCD indicates that the prevalence of children meeting the diagnostic criteria for PANS among this group may be as high as 5% (Jaspers-Fayer et al., 2017).

The onset of PANS often occurs during prepuberty, with boys being twice as likely to have the condition (Calaprice et al., 2017). The symptoms typically have an episodic course, in which they relapse and appear in periods (Gromark et al., 2021; Johnson et al., 2019). While some experience remission and may not have any symptoms for at least a year, others experience a chronic-static course, with symptoms stably present over a longer period of time (Gromark et al., 2021).

The aetiology of PANS is still not clearly understood (Chang et al., 2015). However, research to date suggests that there is a correlation between some types of infectious triggers—such as streptococcal infections, Lyme disease, mycoplasma, and influenza—and the appearance of symptoms (Chang et al., 2015; Swedo et al., 2012). This, together with the high representation of allergies as well as inflammatory conditions such as eczema and asthma in children with PANS, suggests a possible underlying immune dysregulation as an aetiology of the disorder (Calaprice et al., 2017; Johnson et al., 2019).

Clinical management of PANS includes anti-inflammatory medication and other immunological interventions, such as intravenous immune globulin (IVIG), to target inflammatory processes (Frankovich et al., 2017), and various antibiotics to treat and prevent infections (Cooperstock et al., 2017). In addition, psychological, behavioural, and psychopharmacological interventions are also suggested to decrease suffering and improve adherence to therapeutic interventions (Thienemann et al., 2017).

The procedure for diagnosing PANS still lacks consensus, and some particular challenges in diagnosing it have been noted by researchers (Chang et al., 2015). Firstly, by definition, the PANS symptoms overlap with a variety of psychiatric disorders, such as OCD, Tourette syndrome, ADHD, depression, and bipolar disorder, making PANS difficult to identify (Chang et al., 2015). Secondly, due to the abrupt onset, the inherent neuropsychiatric symptoms can be misinterpreted as a reaction to acute psychosocial trauma (Chang et al., 2015). Thus, it is a condition that can be overlooked and thereby left untreated.

Despite the unpredictability and severity of PANS only a few studies of date have been conducted with the aim of understanding the experience of being the parent of a child with the condition (Farmer et al., 2018; McClelland et al., 2015). In a study conducted in the US, McClelland and her colleagues (McClelland et al., 2015) interviewed approximately 120 family members of individuals with PANS, most of them parents, to explore their experiences of living with the condition. The study identified three themes. The first involves the experiences of intense fear of the symptoms: Participants were terrified by the symptoms themselves. The second theme is related to the frustration regarding lack of scientific knowledge about the disorder, its causes, and which treatments are effective (McClelland et al., 2015). The third theme describes the perception of not being heard by members of the healthcare community, as well as the feeling of being blamed for causing the symptoms, or being met with scepticism (McClelland et al., 2015).

In another study, also conducted in the US and using the Caregiver Burden Inventory, Farmer et al. (2018) investigated parents’ perceived burden of caring for a child with PANS. Study participants consisted of approximately 100 parents of children 4 to 18 years of age who were diagnosed with PANS. Results indicated that parents perceived the burden of caring for their child as significantly higher, in comparison not only to parents of healthy children but also to parents of children with other psychiatric conditions (Farmer et al., 2018). More specifically, parents perceived that the symptoms had an negative impact on how they managed daily routines and the demands of everyday life (e.g., needing to guard their child around the clock), on their personal development (e.g., feeling that they are missing out on life), on their physical health (e.g., not getting enough sleep), on their emotional health (e.g., feeling shame and guilt), and on their social relationships (e.g., having marital problems; Farmer et al., 2018). In addition, the findings revealed that half of the children had been forced to switch schools, and parents who reported that their child missed at least one day of school per week due to PANS symptoms reported significantly higher perceived burden than those who did not (Farmer et al., 2018). Furthermore, mothers who had to reduce their work hours as a consequence of their child’s illness also reported significantly higher parental burden (Farmer et al., 2018).

In another US based study, using the Caregiver Burden Inventory, Frankovich et al. (2018) investigated the relationships between various medical and demographic factors and parents’ perceived burden regarding having a child with PANS. The results showed that severity of PANS, assessed by clinicians, was significantly related to parental burden (Frankovich et al., 2018). In addition, it was found that a shorter time between symptom onset and the first appointment at the PANS clinic was related to a lower perceived burden (Frankovich et al., 2018). On the other hand, the time between the first appointment at the clinic and when medical intervention was given did not predict parents’ perceived burden (Frankovich et al., 2018).

Parental stress is a distinctive type of psychological stress that arises when parents’ perception of parenting demands is greatly higher than their perceived resources for managing these demands (Deater-Deckard, 1998). Parental stress is “role specific”, and is a distinct reaction to the specific demands associated with parenthood (Deater-Deckard, 1998).

The current study aimed to generate an understanding of aspects related to parental stress in parents of children with PANS. More specifically, we aimed to explore: (1) parents’ appraisals and emotions related to their child’s symptoms; (2) parents’ appraisals and emotions related to healthcare providers; and (3) parents’ appraisals and emotions related to managing problems related to their child’s illness.

Generating an understanding of how parenting a child with PANS affects parental stress is important not only because it has consequences for parents’ life in general, but also because it may have crucial implications for parents’ ability to manage their child’s illness (Ryan & Sawin, 2009). As PANS is still a relatively unknown and misunderstood illness, the results of such an investigation are particularly useful for healthcare professionals who work with these children and their families, as well as for informing future research.

## Methods

### Design

The present study employed in-depth semi-structured individual interviews with parents of children diagnosed with PANS. The choice of applying in-depth semi-structured interviews was the result of the intention to explore individual first-hand perspectives as expressed by the parents themselves. Individual interviews allow to conduct an open exploration of the complexity of the person’s appraisals, behaviours and emotions (Kvale & Brinkmann, 2009).

The study was reviewed and approved by the Swedish Ethical Review Authority; approval number 2020–06554.

### Recruitment procedure and participants

The recruitment of parents of children diagnosed with PANS was done via an announcement on the websites of patient organizations, and in waiting rooms at child and youth medical clinics in the city of Stockholm. In the announcement, in addition to the provision of general information about the study, parents were encouraged to contact the first author via email or telephone. Interested parents received further written information about the study and terms of participation. A total of 13 parents contacted the first author to receive information about the study, and all of them agreed to be interviewed. Participants were not offered compensation for their participation. Inclusion criteria were parents of children aged seven to 19 years who had been clinically diagnosed with PANS.

Variation was found among participants in terms of the sex of parent and child, the child’s age as well as reported time of diagnosis, the course of the symptoms (i.e., episodic or chronic-static), and treatment experiences. (Table 1) describes the participants’ characteristics.

**Table 1.** Participants characteristics

Characteristic	
Female/male	8/5
Child’s age at time of interview Mean age ± SD (years) Median age (years)	12.9 ± 2.6 12.5
Child’s age at time of first flare Mean age ± SD (years) Median age (years)	9.9 ± 3 10
Child’s age at diagnosis Mean age ± SD (years) Median age (years)	9.9 ± 3 10
Course of symptoms Episodic course Chronic-static course	9 4
Treatment experiences Antibiotics Intravenous immune globulin (IVIG) Anti-inflammatory medications Psychiatric medications Cognitive behavioural therapy	13 9 12 12 13
Parents’ living arrangements Two-parent home Single-parent home Joint physical custody*	7 4 2
Siblings No siblings Have siblings	2 11

*Children living alternatively and approximately equally with each parent after a parental divorce.

### Data collection

In order to obtain rich descriptions of parents’ appraisals, emotions, and behaviours in relation to having a child with PANS, a semi-structured interview guide was created, and follow-up probing questions were used to further explore aspects identified in previous interviews. (Table 2) outlines a sample of interview questions.

**Table 2.** Sample of initial interview questions

Initial Open-ended Questions
Tell me about your child’s PANS. What does his/her PANS mean in terms of behaviours/symptoms? Tell me about your family. Your work situation? Tell me about what your child’s PANS looks like. Does it come and go? Is it the same? What do you think about it/how do you feel about it? What does it mean to you? Tell me about a regular day in your family life.
*Intermediate Questions*
Tell me about specific everyday activities that are challenging. What do you do? What do you think? How do you feel? What do you think about your behaviour? Tell me about the first time your child showed symptoms. What was it like? What have you thought and how have you felt? What did you do? Tell me about the second time your child showed symptoms. How did you feel? What were your thoughts? What did you do? Tell me about a specific situation that you think works well with your child. What does it look like? What do you do? What do you think and how do you feel? Tell me about what medical treatments your child has received. What led to the treatment? How do you perceive the treatment? How do you perceive the healthcare providers with whom you and your child have had contact? Tell me about when your child received the PANS diagnosis. What led to the diagnosis? How do you feel and what do you think about it? In what ways has PANS effected your family? What do you do/how do you feel/what do you think in such situations? In what ways has PANS affected you? What do you do/how do you feel/what do you think in such situations? Is there someone who gives you support? In what ways do you receive support? *Concluding Questions* What do you think about the future? What do you think will help you and your child? Is there something else you think I should know in order to understand what it is like to be the parent of a child with PANS?

Data collection took place between February and July, 2021. The interviews, conducted at the participants’ homes, lasted 40–100 minutes. All interviews were conducted, audio-recorded, and transcribed by NR.

### Data analysis

The aim of data analysis was to attain a condensed and broad description of aspects related to parenting stress among parents to a child with PANS. An inductive qualitative content analysis approach suggested by Elo and Kyngäs (2008), was used as a guide for the analysis of data. The reason for applying this method of analysis is that it is a content-sensitive method which enables researchers to explore the meaning of communication, and to identify processes concerning individual’s meanings, intentions, consequences and context (Elo & Kyngäs, 2008), what was suitable with regards to the aims of the study. In addition, not restricting the analysis to predefined categories, is particularly appropriate for this study as the research on parents’ experiences of having a child with PANS is limited and to date no studies have been conducted regarding parents’ management strategies.

The data analysis focused on the manifest content and involved three stages: open coding, creating subcategories and abstraction (Elo & Kyngäs, 2008). In the first stage, the open coding, the data was broken up into its components, parts, and properties, and defined by the actions and meanings described by participants. Units of meaning were coded by the semantic content of them, and was done with the help of questions regarding the participants’ actions, appraisals and emotions. These questions included “What process is at issue here?”, “How does this process develop?”, and “How does the participant think and feel while involved in the process?”. This stage results with codes that together describe all aspects of the content. The open coding of all interviews was conducted by NR.

In order to increase the trustworthiness of the open-coding analysis, the data interpretation was checked and discussed at a seminar with five researchers with extensive experience of qualitative research. Prior to the seminar, participants received transcriptions of two interviews and the open coding protocol. During the seminar, the coding was discussed and refined. The rest of interviews were analysed based on these codes.

In the second stage of the data analysis, the creating of subcategories, codes were sifted, organized, and synthesized into higher order headings. Grouping of codes involved comparative methods and searching for similarities and differences between codes. Grouping of codes was done not only in relation to what makes a code a part of one sub-category, but also in relation to what makes it not being a part of another (Elo & Kyngäs, 2008). Subcategories were named with direct citations from the interviews.

In the third stage, the abstraction, subcategories which described components of similar aspects related to parenting stress were grouped together, rubric were chosen based on content-characteristic. The creating of subcategories and the abstraction, was conducted by NR and LPR together, in repeated meetings. The data analysis was supported by QSR International’s NVivo 10 software.

To increase trustworthiness our personal positioning in the course of carrying out this research was subject to reflection during the whole research process.

## Results

The analysis of interviews resulted in five identified categories, which together reflect aspects related to parental stress in parents of a child with PANS. The first three categories reflect parents’ appraisals related to the condition: “Being effected by PANS”, “Experiencing PANS over and over again”, and “Having no control”. The fourth category, “Obtaining medical treatment is challenging”, entails appraisals related to contact with clinicians. The fifth category, “Managing problems”, consists of strategies parents apply in order to manage problems related to having a child with PANS. (Table 3) summarizes the identified categories and subcategories. Citations from interviews are used for illustrating the content of subcategories. In order to protect confidentiality of subjects neither gender nor names are used instead referred to as number of transcript.

**Table 3.** A summary of identified categories and sub-categories

Category	Subcategory
Being effected by PANS	She was a completely different child
	His suffering is unbearable
	I wasn’t a mother to my other children
	I vanished as a person
Experiencing PANS over and over again	The first time it happened I was in shock
	And then she had another serious flare again
	It is a war against time
Having no control	She really had to do it
	There was never anything that worked
	I can never predict how she’ll be
	The only thing that would help is medicine
Obtaining medical treatment is challenging	There is no flexibility
	It’s hard to get a continuous and clear care plan
	We, parents, are blamed the entire time
Managing problems	I had to hold her many times
	We divide up between us
	It’s almost as if I don’t see it
	As soon as she starts feeling better, it’s “Out!”
	Those who’ve helped me, that’s the mothers’ group
	I read an article in some neuroscience journal
	I wrote my own referral to the doctor

### Being affected by PANS

According to the parents, a prominent part of the experience of having a child with PANS is related to the radical and intense negative consequences of the symptoms. All parents perceived the symptoms as intense and overwhelming, with a total impact on the child, everyday life, and the parent him/herself. This category consists of four subcategories, each one of them expressing a different type of consequence.

#### “She was a completely different child”

Many of the parents perceived that the most difficult experience of PANS was the total change in their child’s personality. The change was often described as seeming as if their child had been replaced by another one or as losing a child and receiving a new one, as expressed by one of the parents in the following:

*That might have been the biggest shock in the beginning: She was always happy, unbelievably creative, she could always find solutions. I couldn’t imagine her being depressed or sad or not wanting to live – that didn’t exist … She was like a completely different person; [it was] like my daughter disappeared. (Transcript 1)*

Several parents described longing for the child they had previously known, and experiencing grief at losing the child after he/she became sick.

#### “His suffering is unbearable”

The symptoms of PANS were described by all parents as causing enormous suffering for the child. They described the suffering their child experienced as emotional (such as extreme and intense anxiety) and physical (such as not having the energy to leave their bed for days), as well as in terms of losing skills, abilities, and interests, and the loss of their childhood. The child’s suffering had led to sorrow and emotional pain in the parents, and was perceived as a major source of stress, as expressed in the following:

*He’d been wiped out. Had compulsions 24 hours a day … And the suffering. The suffering was the worst. And it’s three years of his life that have disappeared. To be forced to live in his body when he didn’t actually want to be there – that makes me hurt so much inside. (Transcript 6)*

#### “I wasn’t a mother to my other children”

Parents perceived negative consequences of the condition on the everyday functioning of the entire family. In everyday life, taking care of the sick child made it difficult to satisfy the family’s basic needs, such as buying groceries and preparing food. Parents also perceived that they could not satisfy the psychological needs of the child’s siblings, or build relationships with them as they wished to. PANS had consequences for siblings, not only because the sick child needed the parents’ care and attention but also because the siblings often needed to adjust to the needs of the sick child. In addition, in some families, siblings suffered from the sudden violent behaviours of their sibling with PANS. Experiencing the negative consequences of PANS on the child’s siblings caused parents to have negative emotions of self-blame, frustration, and sorrow. This perception is expressed in the following:

*We adjust ourselves a great deal based on how he feels and what he’s able to do. And there’s something that we parents suffer a lot from: that it has consequences on the siblings. We don’t have friends who come over because it’s not sustainable, and that affects his siblings a great deal. I think that’s tough. (Transcript 4)*

#### “I vanished as a person”

All parents perceived that the intensity of the symptoms, and taking care of their sick child, many times forced them to neglect their basic needs, such as sleeping and eating. Other needs related to interests, personal development, and social relations were also ignored due to both taking care of the child and a lack of energy, as one of the parents describes in the following:

*I vanished as a person; I was forced to completely focus on my child and to manage the everyday. So my needs, they had to take a back seat for so long that in the end I didn’t know who I was. In the end I had no needs – it was as if they’d disappeared. I knew that, my breath, no one can take that away from me – but everything else was taken by her illness. (Transcript 1)*

### Experiencing PANS over and over again

The process of understanding that PANS is a chronic condition, with the overwhelming, terrifying, and confusing effect of the first flare, followed by the sorrow related to the second flare, to the perception that the child’s condition becomes worse with every flare, all contribute to parental stress.

#### “The first time it happened i was in shock”

When describing the first time they noticed their child’s condition, all the parents could describe in detail the time and context of the event. The symptoms had often appeared with no warning signs, leaving the parents in shock. When the first flare disappeared, the parents were often left with mixed feelings of not only relief but also confusion at not having an explanation for their child’s behaviour, and fear that it might happen again. Several parents described the fear related to the first flair as being so intense that they avoided places and people that may remind them of the event, and had intrusive images and dreams related to it.

#### “And then she had another serious flare again”

After having the symptoms for the first time, some children recovered without receiving any specific medical treatment while others were given psychopharmacological medications, a few receiving antibiotics or anti-inflammatory medication. Regardless of what had led to the end of the first flare, the reappearance of the symptoms with the second flare was perceived as a challenging experience, entailing sorrow and hopelessness. According to some of the mothers, the realization that the symptoms were not just a one-time phenomenon made them especially vulnerable to becoming depressed. This is how one of the parents described the experience:

*And then she had another serious flare again. Then it felt horrific. That she’d been doing so well, and we’d hoped that we’d be able to a new apartment we wanted – maybe we can do what we want. Then it all completely crumbles again. (Transcript 2)*

#### “It is a war against time”

A source of stress for parents was the appraisal that every flare would lead to irreversible damage in the child’s brain, causing a loss of abilities so that the child’s condition would grow increasingly worse with every flare. The perception of an irreversibility of the damage to the child’s brain functioning was described as a “war against time” and a “ticking bomb”.

### Having no control

Parents experienced that they did not have the ability to control, prevent, or even decrease their child’s condition. In the analysis of the data we identified three components related to this lack of control: the unpredictability of the symptoms, the forcefulness of symptoms, and the experience of lacking effective techniques.

#### “She really had to do it”

Parents perceived that the origin of the condition was in their child’s brain, and experienced that their child had no control over them. PANS was stronger than their child, was a result of a disfunction, and was not related to the child’s will or motivation.

#### “There was never anything that worked”

Parents described putting enormous effort into trying different techniques and approaches to prevent their child’s symptoms or at least decrease their intensity, but discovered that such techniques had no effect. The symptoms exist, and there was nothing to do to stop them. The inability to prevent the symptoms was associated with feelings of helplessness, lack of power, and frustration. As one of the parents said:

*What was frustrating to me was that all the methods we used then – all the methods I learned, strategies and response – they didn’t work on my child. And the school experienced the same thing. They tried their very best, but there was never anything that worked. And that was really frustrating. (Transcript 10)*

#### “I can never predict how she’ll be”

Interestingly, both parents whose child had PANS of the more episodic type and those whose child’s symptoms were more stable experienced that they could not predict the state of their child from day to day. In addition, many parents experienced that they could not discern a pattern in which their child’s flares appeared; when flares came and went, or what could trigger a flare. The experience of not being able to predict the flares resulted in the parents being poised to react even in times of relatively few symptoms, as one of them expressed:

*For me, it feels like I can never know how it’s going to be with him. I’m always tense and prepared for the possibility that it might be different soon. It’s not about being good at reading him; it just comes like a flash. It’s there the whole time – it’s hard to know what will trigger him. It’s impossible to know what will make it better or worse. (Transcript 11)*

#### “The only thing that would help is medicine”

All parents perceived that pharmacological treatment was the only way their child could be cured of PANS. Although psychological and educational interventions may have had an effect on other problems the child may have, the PANS symptoms could only be eliminated or decreased through medication. Several parents perceived the effects of antibiotics in terms of a miracle, and were amazed at the rapid positive improvement in their child’s functioning after receiving the treatment. Indeed, the length of time that had passed since a pharmacological treatment was a factor that all parents attributed to their child’s functioning.

### Obtaining medical treatment is challenging

Experiences related to receiving healthcare and being in contact with healthcare providers were generally a source of negative emotions, such as frustration, helplessness, desperation, and hopelessness. Some parents, however, talked about positive experiences with healthcare providers. The following category is constituted of subcategories of parents’ perceptions related to the healthcare the child and the parents receive.

#### “There is no flexibility”

A major struggle parents had in relation to their child’s healthcare involved the clinic’s and healthcare providers’ lack of knowledge, leading to inflexibility and a lack of appropriate support. Inflexibility was experienced in several ways. Firstly, it was experienced in relation to how clinicians understood the symptoms and their aetiology. Parents experienced that clinicians often held onto a certain type of model of explanation for the symptoms rather than trying to understand them from different perspectives. One of the parents said:

*Just because my son has an autism diagnosis, every time somebody in healthcare opened his journal the reaction was “No, it’s his autism that’s acting up”. And no one’s ever listened to me when I’ve said it could be his [PANS]. And both the children’s psychiatric clinic and occupational therapy just said “No, he has autism; you’ll have to live with this”. (Transcript 11)*

As the quote illustrates, the parent experienced rigidity in the way clinicians understood her son’s behaviours, seeing them as manifestations of his autism and proposing only one type of healthcare based solely on this assumption.

Experiencing rigidity from healthcare providers was also related to procedural and practical aspects, such as the time and place of appointments. For example, many parents experienced that their child’s symptoms made it difficult to visit the clinic or to have appointments early in the day. When they asked for adjustments to meetings, they were met with inflexibility. Such rigidity constituted a cause of stress in parents.

#### “It’s hard to get a continuous and clear care plan”

All parents experienced that there was unpredictability and discontinuity in the treatment their child received, never knowing what the next stage of the treatment would be or how long the current treatment would last. Many parents described their child’s treatment in terms of luck or coincidence. Parents’ experiences of not having a given, known treatment plan caused them to have feelings of anxiety, tension, and helplessness, as illuminated in the following quote:

*It’s hard to get a continuous and clear care plan. It’s always that way when the last treatment’s given – then we don’t know what’s going to happen now. The whole time we experience this uncertainty, the whole time. I get very frustrated. (Transcript 6)*

#### “We, parents, are blamed the entire time”

In their journey, parents perceived that they were blamed or criticized for their child’s condition or for not being able to follow clinicians’ demands. Parents perceived that clinicians might write complaints about or criticism of them in their child’s medical journal, which made them afraid. To avoid this risk, parents were careful not to say anything that would provoke or irritate the medical staff. In addition, experiences of being blamed led to feelings of hopelessness and self-criticism, and was a cause of stress, as one of the parents describes in the following:

*There’s always an accusation of us parents. If she’d been wearing long socks she’d have slept better. If you’d cut off the Wi-Fi she’d have been happier. There’s always an accusation. We never leave there without getting a lecture on what we should’ve done. When the child’s that sick, it’s extremely hard to hear that. They don’t focus on the child. They focus on us. You two, what are you doing wrong? And if the child’s that sick, you don’t have the energy for accusations. (Transcript 1)*

### Managing problems

This category contains parents’ behaviours, appraisals, and emotional experiences that describe their attempts to manage various problems related to their child’s PANS. These attempts were categorized into seven subcategories, each representing a different type of management and/or targeting a different problem.

#### “I had to hold her many times”

For a large part of the day, parents were engaged in activities related to preventing physical harm to their child. This was done both by immediately and physically stopping the child from hurting her/himself, and through different strategies for minimizing the long-term harm caused by the symptoms. For example, one of the parents bought a particular moisturizing hand soap for her child to minimize the damage caused by his compulsion to wash his hands. This type of management is related to parents’ appraisals that the symptoms were stronger than the child, the unpredictably of the symptoms, and not having an effective method to prevent them. This type of management led to the short-term prevention or minimizing of problems, but forced parents to be constantly alert and poised to react, as the following quote illustrates:

*She had a compulsion to run into the road; she tried to hit strangers in town. So I had to hold her. I had to hold her many times. I was in such stress to protect all the things and protect her constantly. (Transcript 2)*

#### “We divide up between us”

In order to manage the negative consequences that the symptoms had on siblings, some parents tended to divide the family, doing separate activities with the siblings. Dividing the family was a strategy that was possible only when there were two parents at home or if the parent received help from another adult. Many of the parents who used this strategy felt sorrow at not being able to do activities as a whole family, as they believed that family togetherness is an ideal to strive for. Separating the child from his/her siblings, however, was a way to be able to “be a parent to the other child”, showing interest in and being attentive to the sibling. This strategy is described by one of the parents as follows:

*What helps us is that we divide up between us. We’ve even had a schedule. Planned who’ll go with whom. Dividing ourselves between the siblings has to do with protecting them. (Transcript 6)*

#### “It’s almost as if i don’t see it”

Some parents experienced that when the child’s suffering was unbearable, overwhelming, or difficult to understand, they became less conscious in the situation and grew emotionally detached. In this way the fear and sorrow became more manageable, as one of the parents described:

*When there are symptoms like that, that are very difficult and that I don’t really understand, it’s hard to absorb how bad it is so it’s almost as if I don’t see it. She has such a hard time that I don’t have the energy to put myself in it and see it and absorb it. (Transcript 2)*

#### “As soon as she starts feeling better, it’s ‘Out!’”

During periods in which their child felt better and the symptoms were less aversive, parents put special effort into compensating for “lost time”. This involved doing more enjoyable activities, appreciating nature and music, being social, or even working more. This strategy was a way to manage the emotional exhaustion they often felt, as well as the feeling of losing oneself as a person.

Compensating for lost time also involved the child, as many parents tried to create more enjoyable moments with the child when he/she was not in a flare, giving back “the lost childhood” and compensating for his/her suffering. Compensating for lost time was a strategy that some parents consciously used, while others had developed the strategy without reflecting on it. However, this strategy was available only to parents whose children had periods with milder symptoms. Parents whose children had a more stable manifestation of symptoms did not have this possibility, as the following quote illustrates:

*In recent years it’s gotten more static, sort of; it hasn’t gone in flares. Before, there were flares – it went down down down, and then started getting a little better; there was a clear improvement, it was manageable. Now it’s bad more on a stable basis. It’s more difficult to never have any time to come up for air … When you can relax and live normally a bit. (Transcript 12)*

#### “Those who’ve helped me, that’s the mothers’ group”

Seeking, and accepting, support from others is another strategy parents applied in order to manage problems they had in relation to their child’s PANS. The analysis of the interviews suggests that there might be a difference between mothers and fathers in the ways they seek support. While all mothers described a purposeful and active strategies to find social support outside the family, none of fathers described such strategies.

Mothers sought support mainly from informal social networks such as other family members, usually their own parents, or from other mothers of a child with PANS, whom they obtained contact with via social media. Support could be both emotional (being encouraged or understood by others) and practical (learning about PANS or receiving information about treatment possibilities). The following quote illustrates how one of the mothers made a strategic decision to seek support, and how she received it from another mother of a child with PANS:

*In all these years I’ve learned that it’s in these private Facebook groups that there’s help available … And after a number of months I see how people write. And I feel like, this mother, we hit it off. So I wrote to her: “Want to be my friend?” And we started writing to each other a number of times a day. All the time. We write about how we feel, how we can’t stand it anymore. In some way we cheer each other on and give each other understanding. (Transcript 8)*

However, two mothers, whose child had been diagnosed with PANS for a relatively short time, preferred not to seek and receive support from other parents of children with PANS for fear of recognizing themselves, their child, or their situation in others. For these parents, others’ recognition felt threatening rather than encouraging.

#### “I read an article in some neuroscience journal”

In their management of problems related to receiving medical treatment for their child, parents actively sought scientific knowledge about PANS and treatment possibilities. Seeking knowledge could entail reading academic articles and participating in scientific conferences. Interestingly, this management strategy was expressed by all parents, regardless of their level of education or profession. Becoming an expert was described as a necessity in order to be able to get the medical treatment the parent believed was most effective, so that one could argue for this treatment when meeting with the healthcare provider. This management strategy is described by one of the parents:

*So I called the clinic and said that we have to try something else. Kåvepenin wasn’t helping him anymore – can we have Spektramox? It’s broader, and many people experience better effects from it. I was the one who suggested the medicine. You have to read up on it. Even though we have very good doctors, we have to educate ourselves so we know what alternatives there are. (Transcript 7)*

#### “I wrote my own referral to the doctor”

Another strategy that was used to manage problems related to receiving medical help for the child involved actively and purposefully searching for medical clinics and practitioners others had recommended. Searching actively and approaching specific clinics also meant that, in some cases, parents concluded their contact with the clinic when they were not satisfied with the healthcare their child received. The following quote demonstrates this strategy:

*We go from one doctor to another, but not randomly; I think “Okay, we’re not getting help here – what else is there?” I ask other parents where you can find help. Then I got a tip about this doctor who’d started being interested in PANS. (Transcript 9)*

## Discussion

### Discussion of the results

PANS is a relatively new diagnosis, and the condition’s aetiology, treatment, outcomes and prevalence are not clearly understood (Chang et al., 2015; Gromark et al., 2021). The current study aimed to generate an understanding of aspects related to parental stress in parents of children with PANS. The applied qualitative methods allowed us to openly explore parents’ experiences from the perspectives of the parents themselves. The analysis of interviews identified five categories of parents’ experiences related to the radical and intense negative consequences of PANS, the chronicity of the condition, the lack of control over the symptoms, the challenges in obtaining medical treatment, and the strategies parents used to manage problems.

According to the Transactional Theory of Stress and Coping (Lazarus & Folkman, 1984), in order to understand psychological stress related to an event we need to understand the individual’s appraisal of the event, as well as how the individual perceives the event’s manageability. The theory suggests that people constantly engage in interpreting events in their environment in order to evaluate the threat or potential harm they pose to their well-being (Lazarus & Folkman, 1984).

These interpretations are based on two types of appraisals: appraisals related to the nature and characteristics of the event, for example, how familiar or ambiguous the event is to the individual, and appraisals related to the individual’s personal agenda, values, goals, beliefs, wishes, expectations, and personal needs (Lazarus & Folkman, 1984). Based on these appraisals, the individual evaluates the event as insignificant, positive, or threatening (Lazarus & Folkman, 1984). Interpreting an event as threatening will lead the individual to engage in appraisals related to one’s perceived ability to deal with the threatening event (Lazarus & Folkman, 1984). The individual identifies their possibilities to manage the threat by evaluating their personal resources, ability, skills, ways in which the individual has coped with similar events in the past, and characteristics of the situation, such as demand and resources in the environment (Lazarus & Folkman, 1984).

Looking at the results through the Transactional Theory- considering the tremendous negative effects of PANS, the chronicity of the condition, appraisals of having no control, and having difficulties to receive medical treatment- suggests that parents of children with PANS are particularly vulnerable to experience high levels of psychological stress.

Similar to previous studies (Farmer et al., 2018), the negative consequences of the symptoms were identified as an aspect that contributed substantially to parental stress. As also found in previous studies, parents perceive the episodic and explosive onset of symptoms as frightening (McClelland et al., 2015). More specifically, the current study identified that the characteristic of the symptoms that involved causing the child to suffer was also related to a radical change in the child’s character. Parents describe feeling as if they had lost one child, which was replaced with totally different child. This experience had a contributing effect to parents’ psychological stress.

Confirming findings from other studies (Farmer et al., 2018; Frankovich et al., 2018), parents perceive a negative impact of PANS on their social relationships with others. Findings illuminate the effects PANS has on relationships within the family, as well as parents perceived reduced ability to function as parents to their other children, which constitutes a particular source of parental stress.

In addition, the current study identified specific appraisals related to PANS as being a chronic condition, suggesting that the appearance of symptoms a second time, after a period without symptoms, is a particularly vulnerable period in terms of parental stress. Additionally, the perception of an irreversibility of the damage to the child’s brain caused by each flare was identified as a stressor.

The results demonstrate that parents in the present study perceive an inability to control the symptoms, and feel helpless when it comes to having a method or technique that might enable them to prevent or decrease PANS in their child. Furthermore, the combination of the perception that the only way to treat PANS is through pharmacological treatment and the perception that this is challenging also led to feelings of helplessness, and increased parental stress. Specific challenges in relation to receiving medical treatment are the perceived inflexibility of clinicians as well as the unpredictability of the course of treatment.

Similar to the study by McClelland et al. (2015), the current study has shown that parents felt that healthcare providers blamed them for their child’s symptoms, an experience that leads to negative emotions and increase stress in parents.

The analysis of parents’ strategies for managing problems related to their child’s PANS illustrates that parents use emotional, cognitive, and practical strategies as well as social support. These strategies, however, do not aim to have an impact on the symptoms themselves but rather to decrease their negative consequences. To prevent harm to their child due to the lack of control over their symptoms, parents guard their child and stop them from harming themselves.

Other strategies used for managing the negative emotions parents experience in relation to their child’s suffering included distancing self upon the appearance of symptoms, focusing on the positive things, and compensating for the child’s suffering during periods between flares. These strategies, again, aim to minimize the negative effects of the symptoms rather than to achieve control over them.

Two management strategies, however, aimed to decrease problems related to receiving medical treatment: actively seeking and acquiring scientific knowledge about PANS and treatments; and actively and purposefully seeking clinicians recommended by others. Actively seeking informal social support from other parents of a child with PANS aims both to mitigate negative emotional consequences in parents and to target the illness itself, as social support also includes practical knowledge about treatment options. With regard to this last aspect, the analysis suggests that there might be a difference between mothers and fathers, with mothers being more active in their search for social support while fathers do not use this management strategy.

### Methodological considerations

The present study has some methodological limitations which need to be addressed. Firstly, the study consisted of a small group of parents, the majority of them are members of the same patient organization. Due to limited access to participants, all parents who were willing to take part in the study were included. Thus, voices of parents with a child with PANS who are not part of an organization or are less active have not been heard. It might be that parents who participated in the study are therefore a selected group which might have influenced the content of the interviews.

Furthermore, all participants were parents of a child who had received a formal diagnosis, had obtained medical treatment for their child, were highly educated, and were active in obtaining medical support. It might be that there are other aspects related to parental stress among parents of children who have not received a formal diagnosis.

With regards to the analysis process, one limitation is that the open coding was conducted only by the first author. Conducting the open coding independently by two researchers would have increased the validity of the results (Elo & Kyngäs, 2008). However, all stages of the analysis, included continuous discussions between the authors regarding the interpretations and categorization of the data.

Another limitation is that questions concerning perceptions of child’s school were not included in the interview guide these aspects clearly play an important role in parental stress, as highlighted in studies conducted in the US, in which children were required to change schools (Frankovich et al., 2018). Understanding the role of school in parents’ experiences is clearly a subject for future studies.

### Implications of the study

The study has both theoretical and practical implications. Theoretically, it contributes to a better understanding of a relatively new research area, where research is still very limited. The study highlights factors that may be related to parental stress among parents of a child with PANS. Particularly, it illuminates how parents’ perceptions of the symptoms, the key role of healthcare providers, as well as parents’ strategies for managing problems related to having a child with PANS, may increase or decrease parental stress.

On a practical level, the study contributes valuable knowledge for healthcare providers to consider in their contact with parents. Firstly, the results indicate that parents see the first flare as a traumatic experience, involving strong feelings of fear and avoidance. Secondly, at the time of the child’s second flare, parents may be particularly vulnerable to feeling hopelessness and developing depression. Thirdly, the study indicates that fathers may be particularly vulnerable to stress as they often lack social support, and may not actively seek informal social support as mothers do. Furthermore, the results highlight the importance of flexibility and predictability when planning a treatment programme for the child, as well as of the inclusion of parents’ perceptions in the process.
